# Critical Issues in the Study of Magnesium Transport Systems and Magnesium Deficiency Symptoms in Plants

**DOI:** 10.3390/ijms160923076

**Published:** 2015-09-23

**Authors:** Natsuko I. Kobayashi, Keitaro Tanoi

**Affiliations:** Graduate School of Agricultural and Life Sciences, the University of Tokyo, 1-1-1 Yayoi, Bunkyo-ku, Tokyo 113-8657, Japan; E-Mail: anikoba@mail.ecc.u-tokyo.ac.jp

**Keywords:** magnesium, MRS2 transporter, chlorosis, Mg deficiency

## Abstract

Magnesium (Mg) is the second most abundant cation in living cells. Over 300 enzymes are known to be Mg-dependent, and changes in the Mg concentration significantly affects the membrane potential. As Mg becomes deficient, starch accumulation and chlorosis, bridged by the generation of reactive oxygen species, are commonly found in Mg-deficient young mature leaves. These defects further cause the inhibition of photosynthesis and finally decrease the biomass. Recently, transcriptome analysis has indicated the transcriptinal downregulation of chlorophyll apparatus at the earlier stages of Mg deficiency, and also the potential involvement of complicated networks relating to hormonal signaling and circadian oscillation. However, the processes of the common symptoms as well as the networks between Mg deficiency and signaling are not yet fully understood. Here, for the purpose of defining the missing pieces, several problems are considered and explained by providing an introduction to recent reports on physiological and transcriptional responses to Mg deficiency. In addition, it has long been unclear whether the Mg deficiency response involves the modulation of Mg^2+^ transport system. In this review, the current status of research on Mg^2+^ transport and the relating transporters are also summarized. Especially, the rapid progress in physiological characterization of the plant *MRS2* gene family as well as the fundamental investigation about the molecular mechanism of the action of bacterial CorA proteins are described.

## 1. Introduction

After potassium, magnesium (Mg) is the second most abundant cation in cells. Numerous physiological processes, such as enzymatic activities and aggregation of ribosome subunits, are Mg-dependent [1,2]. In plants, Mg is the central atom of the chlorophyll molecule. It is generally known that leaves become yellowish when Mg nutrition is limited. However, the molecular basis for this Mg deficiency symptom is not fully understood (for reviews, see Hermans *et al.* 2013 [3]). In addition, unlike the situation with other essential nutrients such as potassium, nitrogen, and phosphorus, it has long been unclear whether there is any interaction between Mg deficiency and the regulation of Mg^2+^ transport. In recent years, some reports have suggested that the system for Mg^2+^ uptake and transport in plants are regulated by external Mg conditions. In this review, critical issues in current studies of Mg^2+^ transport and Mg deficiency are summarized with particular focus on the potential link between them.

## 2. Physiological Features of Mg Deficiency

Long-term Mg deficiency leads to the appearance of Mg deficiency symptoms in leaves. Starch overaccumulation and chlorosis are the typical symptoms of Mg deficiency observed in various plant species [4,5,6,7,8]. More than one week is generally required to produce these two symptoms after the removal of Mg from the nutrient solution. Chlorosis may further reduce the photosynthesis rate and finally lead to growth defects, a condition that can be referred to as late-stage Mg deficiency. To identify the mechanism that produces the Mg deficiency symptoms, step-by-step analysis with time has been performed in several plant species. In the studies, two or three days before chlorosis or other visual symptoms appeared, several impairments including the accumulation of non-structural carbohydrates such as sucrose and starch, reduced photosynthetic CO_2_ fixation, and production of reactive oxygen species (ROS) have been detected [6,9,10,11,12]. Among these defects, the accumulation of sucrose was indicated to be directly linked to the decreased Mg concentration, given that the phloem loading of sucrose through the sucrose transporter requires an adequate Mg concentration [1,7,13,14,15]. Excess carbohydrate has been suggested to suppress *Cab2* gene expression, leading to a decrease in photosynthesis rate [7]. Once photosynthesis activity is impaired, unused light energy could generate ROS, which are assumed to cause photo-oxidative damage to chlorophyll and the chloroplast membrane [16,17].

However, these events, which may occur in the middle stages of Mg deficiency, would not be the only process that results in the leaf senescence. The transcriptome profiling revealed the down-regulation of genes associated with the maintenance of the photosynthetic apparatus as early as several hours after Mg deprivation, which is certainly the early stage of Mg deficiency [18]. Similarly, the down-regulation in chlorophyll production, represented by the decreased expression of the genes encoding the magnesium chelatase subunit H, divinyl chlorophyllide *a* reductase, and other proteins, was seen in the rice leaf three days before starch was overaccumulated (unpublished data). Thus, in addition to the ROS-triggered degradation of the photosynthetic apparatus at the late stage, there could be transcriptional regulation of the photosynthetic activity operating in response to Mg deficiency at the earlier stage. Besides, accumulation of sucrose has not been detected before starch accumulation and chlorosis in the rice plant. Instead, Mg starvation was found to reduce the transpiration rate and inhibit nutrient supply to the source leaf, this was suggested to be the trigger for leaf death [8]. A transpiration defect due to Mg shortage was also found in maize leaves, and was successfully reversed by Mg resupply either to the culture solution or via foliar application [19]. But the severe defect in transpiration activity could not be reversed by addition of Mg to the culture medium, because the resupplied Mg could not be transported to the impaired leaf [8]. The question then is why decreased Mg concentration affects transpiration activity, or probably stomatal closure, in some specific source leaves. One possibility is the stomatal closure via ROS production during the early to the middle stage of Mg deficiency. ROS is the important second messenger leading to the stomata closure in several hormonal signaling including ABA [20]. In fact, Mg deficiency has been implied to have some influences on ABA signaling, which will be mentioned later. Or, the ROS production due to the impaired concentration of metal elements could be another hypothesis. In response to Mg starvation, the amount of several metal elements in leaves can be increased [3,7,10,21], which could cause the metal stress. To define the physiological significance of the transpiration in the framework of the Mg deficiency, it would be useful to determine whether Mg starvation reduces transpiration activity in other plants, such as spinach, bean, and *Arabidopsis*.

Another important question concerns acclimation, or positive response, to Mg deficiency. An increase in the activities of antioxidant defense enzymes has been reported in some plant species under Mg-deficient conditions [21,22]. This acclimation response caused by Mg limitation has been shown to confer Cd toxicity tolerance in rice [23] and in *Arabidopsis* [24]. However, the oxidation state of the key antioxidant molecules including ascorbate and glutathione was markedly elevated in response to Mg starvation after eight days in *Arabidopsis* and 12 days in rice, respectively, [10,23]. Mg-deficiency-induced up-regulation in the leaf antioxidant system does not provide enough protection to Mg-deficient leaves against oxidative damage [25]. To the management against sugars accumulation by Mg deficiency, the expression of the sucrose transporter gene was induced in response to decreased phloem loading activity at the time of sucrose accumulation in sugar beet, [15]. Additionally, up-regulation of both glycolysis and tricarboxylic acid cycle was found in source leaves with excess sugars [5,26,27]. The modification of carbon metabolism found in *Citrus* plants may be another physiological system to cope with the increased requirement for consuming the excess sugars [27]. Comprehensive transcriptomic analysis performed at the early stage (within 28 h) and the middle stage (one week) in Mg-deficient *Arabidopsis* roots and leaves indicated that the progression of Mg deficiency involves the ABA and ethylene signaling network and modification to the amplitude of circadian clock oscillation [10,18]. The potential involvement of the hormonal network and the circadian clock suggests that the influence of Mg deprivation is far-reaching. In the case of ABA signaling, participation under Mg deficiency is suggested to be complex, considering that the ABA-responsive genes upregulated under Mg deficiency included both positive and negative regulators, that the key phosphatase in the signal cascade requires the Mg^2+^, and that the ABA concentration was unchanged [18]. In general, the root is less affected than the shoot by Mg deficiency, which was supported by the transcriptome profiling in *Arabidopsis* [10]. Recently, the proteomics study in the root hair in maize under macro- and micro-nutrient deprivation showed the clear upregulation of many ribosomal proteins particularly under the Mg deficiency [28], indicating the significant impact of the Mg shortage also on the protein synthesis in root hair. It is possible that an important function to cope with Mg deficiency is still veiled in the root.

It should be remembered that the progression of Mg deficiency could be modified by environmental conditions other than Mg concentration. For example, Mg deficiency can be aggravated under high-Ca conditions [29,30,31], or high light intensities [16,17]. Although the decreased Mg concentration is the primary cause of the effects on the plant, when and which symptoms appear may be affected by the experimental conditions and the plant tissue.

## 3. Mg^2+^ Transporters

### 3.1. Mg^2+^ Transporters in Microorganisms

Prokaryotes possess four types of Mg^2+^ transport system. CorA protein is the dominant Mg^2+^ transporter under normal conditions [32]. Other Mg^2+^ transporters are MgtA, MgtB, and MgtE, which are induced in response to Mg deficiency. CorA has two transmembrane segments and is functional as the homopentamer [33]. MgtA and MgtB are P-type ATPases having 10 transmembrane segments [34,35,36,37]. MgtE [38] is a distinct Mg transporter having five transmembrane helices. Expression of MgtA and MgtB is transcriptionally induced under low Mg^2+^ condition through the Mg^2+^-regulated PhoP/PhoQ two-component system [39], and MgtE gene expression is controlled by an Mg^2+^ sensing riboswitch [40]. No homolog of MgtA, MgtB, nor MgtE has yet been found in the plant kingdom, although MgtE shows similarity to the human solute carrier SLC41A [41]. The four Mg^2+^ transporters in *Salmonella* show distinct property for ion transport. CorA is believed to transport Co^2+^ and Ni^2+^ in addition to Mg^2+^, and its ion transport activity is abolished by treatment with Mn^2+^ or cobalt (III) hexammine [42]. MgtE can transport Mg^2+^ and Co^2+^ but not Ni^2+^ [38], whereas MgtA and MgtB transport only Mg^2+^ and Ni^2+^ [42]. In yeast, the essential system for maintaining Mg homeostasis includes five Mg^2+^ transporters belonging to the CorA superfamily; Alr1 and Alr2 are localized in the plasma membrane [43], Mrs2 and Lpe10 are on the mitochondrial inner membrane [44], and Mnr2 is localized on the vacuole membrane [45]. As the name implies, most of the Mg^2+^ transporters were first identified through screening studies aimed to select strains mutated in sensitivity to metal ions including Co^2+^, Al^2+^, and Mn^2+^. The origin of the Mg^2+^ transporters is likely to be reflected in features of the CorA superfamily members in plants, as discussed below.

In the Mg^2+^ transport mechanism of CorA protein, the characteristic tripeptide GMN motif located at the end of the first transmembrane segment has long been believed to play an influential role since this motif is well conserved among a wide variety of organisms, although the conservation of the primary sequences in the CorA superfamily is as low as 15%–20%. In this context, CorA-type transporters are referred as the 2-TM-GxN type [46]. Indeed, the essential role of the GMN tripeptide in ion selectivity was clearly demonstrated by mutagenesis studies showing that single amino acid substitutions in this motif are sufficient to abolish Mg^2+^ transport activity [47,48,49,50]. Recent developments in crystal structure analysis have further provided a unique gating model for the Mg^2+^ transport system through CorA, in which the GMN motif played a critical role for ion selectivity [33,50,51]. If Mg^2+^ is absent from the test solution, CorA can import various divalent cations including Ca^2+^ and Mn^2+^, acting as a nonselective cation channel [49]. Meanwhile, only a small amount of Mg^2+^ introduced to the test solution could block the Ca^2+^ currents with a high affinity (*K*_D_ = 1.6 μM) [49], making CorA a Mg^2+^-selective channel in a physiological condition. As the first step in importing Mg^2+^ through CorA, hydrated Mg^2+^ approaches the extracellular side of CorA protein, and then Mg^2+^ binds to the periplasmic mouth of the pore formed by the GMN motifs with strong electron density [52]. Cobalt hexamine, a structural analog of hydrated Mg^2+^, was shown to block the Mg^2+^ current by a competitive binding around the GMN area [49]. In addition, it is now suggested that CorA acts as a Mg^2+^-deactivated Mg^2+^ channel that can sense intracellular Mg^2+^ concentration through the cation binding site in the cytoplasmic domain where 10 ions can be hosted [49,50,52,53], and operates by a self-regulation mechanism similar to the MgtE gating system [54,55]. As the Mg^2+^ concentration in the cytosol is increasing, the cation binding site can be saturated, which leads the CorA conformation change to the “locked” structure incompetent for transporting Mg^2+^. In contrast, the decreased cytosolic Mg^2+^ concentration results in a recession of Mg^2+^ from the cation binding site, which causes the conformation change to the unlocked state [52]. In this state, the CorA pentamer shows the asymmetrically bended structure and the duration of hydration of the pore is prolonged [52]. In fact, the transmembrane pore of CorA contains highly hydrophobic constrictions and hydration is needed to open the gate [53]. The regulation of Mg^2+^ transport activity by sensing the internal Mg^2+^ concentration is suggested for Alr1 protein in yeast [56,57]. Although the exact mechanisms for the regulation of Mg^2+^ uptake activity in Alr1 have not been determined, it is not likely to be the variation of Alr1 protein accumulation or location [57]. These new findings about the permeability, selectivity, and regulation of CorA family proteins have implications for the study of Mg^2+^ transport and transporters in plants.

### 3.2. Mg^2+^ Transporters in Plants: The Overview

To maintain the homeostasis of Mg in each organelle in the plant cell, specific transporters are believed to function in Mg^2+^ transport across the membrane. Amongst the proteins potentially involved in the Mg^2+^ transport, plant MRS2 family Mg^2+^ transporters can be the most well-investigated proteins (see Section 3.3). Meanwhile, the participation of other transporters in Mg^2+^ transport is possible. Examples are OsHKT2;4 in rice [58] and the SV channel in barley [59], whose Mg^2+^ permeability has been shown by electrophysiology, although OsHKT2;4 and the SV channel are believed to be the dominant transporters of K^+^ and Ca^2+^ ions, respectively, in plant tissues. Non-selective cation channels (NSCCs) are the other candidates for the functional Mg^2+^ transporter. One of the cyclic nucleotide-gated channel (CNGC) family protein, AtCNGC10, has been indicated to mediate Mg^2+^ influx, particularly in the root meristem and distal elongation zones [60]. Determining whether or not the alteration of Mg behavior found in the AtCNGC10 antisense line is directly linked to the function of AtCNGC10 will require further investigation [60]. Voltage-independent NSCC (VI-NSCC) is supposed to catalyze the uptake of several cations including Mg^2+^, Ca^2+^, Mn^2+^, and Zn^2+^ at the resting membrane potentials [61]. Considering that VI-NSCCs are sensitive to gadolinium ion (Gd^3+^), the reduction effect of Gd^3+^ on the ^45^Ca^2+^ flux in *Arabidopsis* root epidermal cells [61] as well as on the ^28^Mg^2+^ uptake in rice root (unpublished data) could be an indication of the significant contribution of VI-NSCCs in the uptake of these ions. The *Arabidopsis* MHX protein is a vacuolar exchanger of protons with cytosolic Mg^2+^ and Zn^2+^ [62]. Preferential enrichment of the *AtMHX* gene was observed in the vascular cylinders of all organs, and accumulation of this protein is regulated at the translation level [62,63]. In this regulation, the 5′ UTR of the *AtMHX* gene, containing 169 nucleotides, has a role in repressing the translation of the coding sequence [63]. The appearance of necrotic lesions in leaves of AtMHX overexpressing tobacco plants grown under elevated Mg^2+^ or Zn^2+^ indicates the critical function of AtMHX in balancing the concentrations of Mg^2+^ and Zn^2+^ in plant cells [62]. However, knowledge about the functions of other MHX proteins are lacking, although the gene is widely conserved in the plant genome [64]. In yeast, the mutant vps5Δ displayed a strong sensitivity to low-Mg^2+^ conditions and was suggested to missort the *trans*-Golgi network Mg^2+^/H^+^ exchanger on the tonoplast [65]. However, the molecular nature of the Mg^2+^/H^+^ exchanger itself has not been identified yet, and no clear homologue of AtMHX has not been found in yeast [65].

### 3.3. Plant MRS2 Family Proteins

The plant MRS2 family belongs to the CorA superfamily and was first identified in *Arabidopsis* by two research groups at approximately the same time, and thus is called either AtMRS2 [66] or AtMGT [67] (Table 1). For simplicity, we refer to it as AtMRS2 in this review.

In the characterization of the function of AtMRS2 transporters in plant, elucidating Mg^2+^ permeability is an important step. For this purpose, plasmid complementation assay using the Mg^2+^ uptake-deficient mutant *Salmonella typhimurium* strain MM281 and the *Saccharomyces cerevisiae* strain CM66 has frequently been performed, and evidence for the Mg^2+^ transport capability of AtMRS2 has gradually increased. Strain MM281, which lacks three genes, *MgtA*, *MgtB*, and *CorA* [38], has provided evidence for the Mg transport ability of AtMRS2-2 [68], AtMRS2-6 [69], AtMRS2-7 [70], AtMRS2-10 [67], and AtMRS2-11 [67]. In contrast, expression of either AtMRS2-1, AtMRS2-10 [71], or AtMRS2-11 [67,71] confers on CM66, an *alr1 alr2* mutant strain of *S. cerevisiae*, the ability to grow and take up Mg^2+^ from medium containing less than 10 mM Mg^2+^. The capability of transporting other metals including Cu^2+^ and Zn^2+^ has also been indicated for some AtMRS2 members [67,70] (Table 1). However these assay systems sometimes yield disparate results. For example, the Mg^2+^ transport ability of AtMRS2-6 was shown by complementation assay in MM281, but not in CM66. Evidence for the Mg^2+^ transport ability of all AtMRS2 family members, including several that failed to confer growth ability on CM66, has been provided by complementation study using the yeast *mrs2* mutant [72]. In this context, the complementation assay using *mrs2* mutant seems to work effectively. Nevertheless, this assay system can only be applicable to the CorA-type transporters in principle [72].

As Mg^2+^ transporters, AtMRS2 proteins are believed to participate in the control of Mg^2+^ concentration in organelles including chloroplasts, mitochondria, and endoplasmic reticulum (ER), as well as the cytosol. In clade B, there are three AtMRS2 members, AtMRS2-1, -5, and -10, which have been well studied (Table 1). Plasma membrane-localized AtMRS2-10 is expressed in the root [72]. AtMRS2-10 has stably exerted the Mg^2+^ transport property in every assay system mentioned above and also can be functionally reconstituted into liposomes derived from *Escherichia coli* without any accessory proteins [73]. Therefore, functions associated with Mg^2+^ uptake in the root have been expected for AtMRS2-10. Overexpression of this gene in tobacco plants caused the increased Mg concentration in plants and contributed to a low-Mg tolerant phenotype and, interestingly, conferred Al tolerance [74]. AtMRS2-1 and AtMRS2-5 are localized to the tonoplast and participate in Mg^2+^ compartmentation to the vacuoles of leaf mesophyll cells under high-Mg^2+^ plus low-Ca^2+^ conditions [29]. The single knockout lines *atmrs2-1*, *atmrs2-5*, and *atmrs2-10* show no phenotype [72,75]. In addition, the double knockout *atmrs2-1* *atmrs2-5*, both of which are localized at tonoplast, shows no phenotype. Interestingly, double knockout *atmrs2-1* *atmrs2-10* shows severe developmental retardation under low Mg^2+^ [31], indicating the redundant system for response to low Mg condition, which is built by MRS2 members localized in the vacuolar tonoplast (MRS2-1) and plasma membrane (MRS2-10) among the clade B members (Table 1).

There are two kinds of AtMRS2 members, AtMRS2-7 and AtMRS2-4, each of which is necessary to survive under low Mg condition. AtMRS2-7 is an ER-localized transporter and its expression in the root is essential for germination in solution culture system as well as for normal growth in low-Mg^2+^ condition [30,72]. However, no modification of *AtMRS2-7* gene expression in response to Mg deficiency has been reported to date. AtMRS2-4/MGT6 had been implied to localize on either chloroplast or mitochondria in shoots [29,72]. However, the recent report has identified AtMRS2-4/MGT6 as a root plasma membrane-localized Mg^2+^ transporter under lowered Mg^2+^ conditions whose transcript levels in the root increased to eight-fold within 12 h of imposition of Mg deficiency [76]. Knockdown of this gene drastically reduced Mg^2+^ uptake activity and consequently reduced Mg content, plant biomass, and chlorophyll content compared with the wild type within four days after the transition to the Mg-deficient condition [76]. This phenotype of the *AtMRS2-4* RNAi plants strongly suggests the important function of AtMRS2-4 in the first step of Mg^2+^ acquisition [76].

AtMRS2-11 is localized to the chloroplast and believed to mediate Mg^2+^ influx, although knockout of this gene has no effect on the Mg concentration in the chloroplast and has produced no apparent phenotype to date [71]. AtMRS2-6 is a mitochondrial Mg^2+^ transporter accumulating particularly in the flower [69,72], and lack of this transporter leads to a defect in pollen development that is also found in the *AtMRS2-2* knockout line [68]. There is little information regarding the role of AtMRS2-3 except its preferential expression in vascular tissues [72].

In monocots, an OsMRS2 family of nine members has been identified in rice (*Oryza sativa* L.) [46,77]. Three of the nine OsMRS2 members do not conserve the GMN motif. However, OsMRS2-4 and OsMRS2-5 carry AMN and OsMRS2-8 carries GIN as the sequence generally corresponding to the GMN motif [77]. The alteration of glycine to alanine, identified in OsMRS2-4 and OsMRS2-5, is exactly the case tested in an artificial mutation experiment to demonstrate the necessity of the GMN motif in Mg^2+^ permeability in the MRS2 protein [78]. The tripeptide GIN has been found in the zinc transporter ZntB, a distant homolog of CorA, and its function is suggested to be the efflux of Zn^2+^ but not Mg^2+^ [79]. The alteration of GMN to AMN and GIN appears to commonly occur in monocot plants [77]. Thus, determination of the molecular function of OsMRS2-4, OsMRS2-5, and OsMRS2-8 may be especially helpful for elucidating the mechanism of Mg^2+^ transport and Mg^2+^ selectivity in the CorA-MRS2-ALR-type proteins, as well as for characterizing the evolution of the plant MRS2 family. To date, Mg^2+^ transport ability has been found for OsMRS2-1, OsMRS2-3, OsMRS2-6, and OsMRS2-9 by complementation assay using the yeast CM66 strain [77]. OsMRS2-6 is suggested to be a chloroplast-localized Mg^2+^ transporter, and the transcription level of OsMRS2-6 in the leaf blade shows diurnal oscillation and is well synchronized with leaf maturation [77]. It is possible that OsMRS2-3 is localized at ER and that OsMRS2-5 is another chloroplast Mg^2+^ transporter, but resolution of this question awaits further study. OsMGT1/OsMRS2-2 is an Mg^2+^ transporter localized at the plasma membrane in both roots and shoots [80], and characterized as one of the ART1-regulated downstream genes [81]. The expression of *OsMGT1* gene is markedly induced within 1 h after Al treatment under acidic conditions. The effect of Mg treatment for reducing Al stress has long been known, and OsMGT1/OsMRS2-2 is now suggested to participate in this Mg alleviation system, given that the knockout of OsMGT1/OsMRS2-2 showed high sensitivity to Al stress that could be rescued by the addition of 10 μM Mg [80,82].

Although knowledge regarding plant MRS2 transporters is increasing, their *in* *planta* functions are still uncertain. The process of Mg^2+^ transport in plants, including root uptake, long-distance transport, and subcellular compartmentation, cannot be described by the MRS2 members and the MHX protein alone. For example, there is only a single MRS2 member, AtMRS2-6, known to localize at the mitochondria in *Arabidopsis*. However, AtMRS2-6 is expressed in very few parts of the plant [69,72], thus hardly seems to bear the Mg^2+^ flux at the mitochondoria in the whole body. Another example is that the cytokinin-reduced P10:CKX3 transgenic *Arabidopsis* showed an increased Mg content, though none of the eight analyzed AtMRS2 genes (AtMRS2-6 was not analyzed) showed increased expressions in the root [83]. In the rice plant, Mg^2+^ uptake is increased in response to Mg deficiency without any change in the expression levels of *OsMRS2* genes in the root (see Section 5).

**Table 1:** 2-TM-GxN type Mg^2+^ transporters in *Arabidopsis* and rice.

Clade	Plant	Name (Number)		Transport Assay		Subcellular Localization	Reference
		MRS2	MGT	MM281	CM66		
A	*Arabidopsis*	11	10	○	○	Chloroplast	[67,69,71,72]
	*Oryza sativa*	6	−	−	○	Chloroplast	[77]
B	*Arabidopsis*	1	2	−	○	Vacuole	[29,66,67,71,72]
		5	3	−	−	Vacuole	[29,66,67,72]
		10	1	○	○	Plasma membrane	[66,67,71,75]
	*Oryza sativa*	1	−	−	○	−	[77]
		9	−	−	○	−	[77]
C	*Arabidopsis*	3	4	−	−	−	[66,67,72]
	*Oryza sativa*	2	1	−	×	Plasma membrane	[77,80]
		3	−	−	○	ER	[77]
		8	−	−	×	−	[77]
D	*Arabidopsis*	4	6	○	−	Plasma membrane *	[30,66,67,72,76]
		6	5	○	×	Mitochondria	[66,67,69,72]
	*Oryza sativa*	4	−	−	×	−	[77]
		5	−	−	×	Chloroplast	[77]
E	*Arabidopsis*	2	9	○	×	−	[66,67,68,72]
		7	7	○	−	ER	[30,66,67,70,72]
		8	8	−	×	(pseudo gene)	[66,67]
		9	−	−	−	(pseudo gene)	[66]
	*Oryza sativa*	7	−	−	×	−	[77]

In the Table, “○” or “×” demote the complementation of the growth defect of the mutant strain, or not. If there is no information, “−” is presented. * Plasma membrane localization was shown in the root cells, while the localization either at the chloroplast or the mitochondria was implied in case of the shoot tissue.

## 4. Mg^2+^ Uptake and Transport in Plants

**Figure 1:**
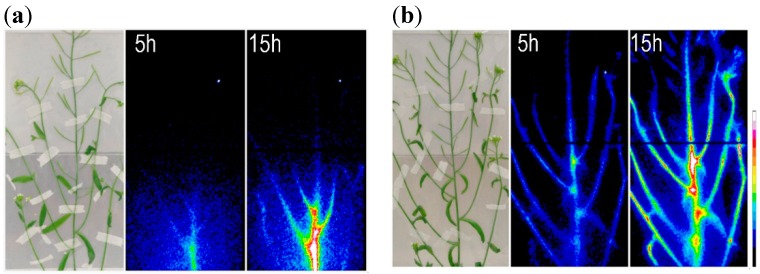
RRIS (real-time radioisotope imaging system [89,90]) captured each radionuclide image at 5 and 15 h of root absorption of (**a**) ^28^Mg; (**b**) ^32^P phosphate. *Arabidopsis thaliana* (Columbia 0) was grown with nutrient solution for 43 days under a light/dark cycle of 16 h/8 h at 22 °C.

Mg^2+^ is considered to be a phloem-mobile element in plants. The Mg concentration in the phloem sap in several plant species showed a similar range; 5.3 mM in barley and 4.9 mM in *Ricinus* [84,85]. The process of phloem loading has been suggested to be strictly controlled, given that the Mg concentration in the phloem sap tended to remain constant even under Mg deficiency or after foliar Mg application [84]. So how does Mg behave in reality? Some parts of the characteristic transport of Mg have been visualized using the radioisotope ^28^Mg (with half-life 21 h). In the rice root, ^28^Mg was found to accumulate preferentially at the root tip soon after absorption from the external solution [86]. In the aboveground part of *Arabidopsis*, the behavior of Mg and phosphate were completely different, at least within 15 h of root uptake (Figure 1). The ^28^Mg gradually flows toward the upper part of the shoot, with steady accumulation in the lower part of the inflorescence (Figure 1). This behavior suggests that Mg^2+^ flows in the xylem vessels while circulating through the cells around the xylem and reaches the upper part of the inflorescence hours after root uptake. Unlike ^28^Mg, ^32^P-phosphate immediately disperses widely in the shoot, especially to the pods and nodes (Figure 1). Similarly, the transport rate toward the shoots of rice seedlings was slightly lower for Mg than for phosphate [87].

The kinetics of the root uptake process in rice has been analyzed isotopically. *K*_m_ and *V*_max_ were estimated as 260 μM and 780 ng·min^−1^ g·[DW]^−1^, respectively, for two-week-old rice plants supplied with normal nutrient solution [88]. *K*_m_ and *V*_max_ of OsMGT1/OsMRS2-2 were estimated as 30 μM and 1.4 μg·min^−1^ g·[DW]^−1^, respectively, using one-week-old rice cultured in 0.5 mM CaCl_2_ solution at pH 4.5 [80]. The difference implies the diversity of Mg^2+^ uptake system in the roots. In addition, the difference might have occurred due to the different conditions of plants. We sometimes found large variation in the uptake kinetic data using the same lines, probably owing to different experimental setups, plant ages, and culture conditions. For example, the Mg^2+^ uptake amount per root volume tended to decrease with plant maturation. Young short roots weighing approximately 20 mg [FW] in one-week-old rice plants showed different uptake kinetics from that in two-week-old rice plants having larger roots of approximately 90 mg [FW], including mature lateral and thick crown roots (unpublished data). Mg^2+^ uptake activity was different in different root segments of the main roots of one-week-old rice seedlings [87], indicating that a heterologous transport system for Mg^2+^ is active even within a single root. Mg^2+^ uptake activity is also affected by the pH of the external solution [1]. In rice, the Mg^2+^ uptake rate from a pH 4.5 solution is twice that from a pH 6.5 solution even during an uptake period of only 15 m [86]. In addition, it is noteworthy that the Mg^2+^ uptake rate doubles within 24 h when a six-day-old rice seedling cultured in 0.5 mM CaCl_2_ solution is simply transplanted to normal nutrient solution for further culture (unpublished data). These characteristic features of Mg absorption in the root should be considered when the Mg^2+^ transport system is investigated, or when the function of each Mg^2+^ transporter is analyzed in plants.

## 5. Potential Regulation of Mg^2+^ Uptake and Transport under Mg Deficiency

In response to the withdrawal of Mg^2+^ from the culture solution, the Mg^2+^ uptake kinetics in rice roots was altered as soon as after 1 h [88]. The alteration has been found to be mostly due to the up-regulation of the high-affinity transport system functioning at low Mg^2+^ concentrations in the external solution [88]. Under this condition, rice seedlings are supposed to sense the Mg deficiency to control the Mg^2+^ transport system. Considering the early response, within hours, the existence of a local signaling mechanism in response to Mg deficiency can be hypothesized. For example, a sudden change in Mg concentration in specific cells might trigger the Mg-deficiency response. In the *Arabidopsis* root, the intracellular Mg concentration in epidermal cells showed more than 60% increase within 10 min of Al treatment as a consequence of a sudden Mg^2+^ influx [91]. Under Mg deficiency, total Mg amounts in both leaves and roots of rice continued to decrease [8] indicating the continuous release of Mg from the root. Given that an adequate concentration of Mg is essential for various physiological activities, a decreased Mg level in the cell due to the shutoff of Mg supply in combination with Mg release under Mg deficiency conditions might contribute to inducing a Mg deficiency response. Nevertheless, the molecular mechanism involved in the induction of Mg^2+^ uptake activity has not been clarified in rice. To date, no induction of any *OsMRS2* gene expression in root has been detected in response to any kind of Mg deficiency treatment (unpublished data). Some regulation of plant MRS2 proteins at the level of translation, modification, including heterologous interactions [92], or gating regulation as described in the prokaryote CorA [50,52,53,93] might be the mechanism controlling Mg^2+^ uptake and transport under the Mg starved condition. Additionally, the participation of other proteins in Mg^2+^ uptake could be considered.

On the other hand, in response to the Mg^2+^ withdrawal from the growth medium, the induction of *AtMRS2-4/MGT6* expression in the *Arabidopsis* roots was reported recently [76]. Interestingly, the expression of *AtMRS2-4/MGT6* in the root of one-week-old seedlings peaked at 12 h after the transition to the Mg starved condition and then subsequently decreased [76]. Then, the fluctuating gene expression may be the reason why the previous transcriptomic studies using five-week-old seedlings could not detect the altered expression of any MRS2 genes [10,18]. Also, the Mg^2+^ uptake analysis we have employed using ^28^Mg in three-week-old *Arabidopsis* has not provided any indication of the up-regulation of Mg^2+^ uptake in response to several days of the Mg starvation so far (unpublished data). Identification of exact condition in which the AtMRS2-4/MGT6 proteins actually function in response to Mg limitations would be essential to further investigations to reveal the transcriptional regulation of this gene. On the other hand the characterization of the *Arabidopsis* mutant lines has provided the knowledge that AtMRS2-4 as well as AtMRS2-7 have the essential role in low-Mg environments since these mutant lines showed reduced Mg content in plants specifically under Mg-limited conditions [30,76], Other than these two molecules, any member of AtMRS2 family including AtMRS2-1, AtMRS2-5, AtMRS2-10, and AtMRS2-11 has not been indicated to have particular functions under the Mg deficiency. In rice plant, retrotransposon Tos17 insertion lines are available only for OsMGT1/OsMRS2-2. The mutant analysis has revealed the essential role of OsMGT1/OsMRS2-2 for alleviating the Al toxicity [80], but there is no information as to whether the knockout of this protein affects the Mg deficiency response in rice.

In case of the leaf, no marked up-regulation in the expression of *AtMRS2* genes [10,18] as well as *OsMRS2* genes (unpublished data) have been observed under the experimental conditions ever tested. Indeed, there has been no indication about the modulation of Mg re-distribution or re-translocation in response to the Mg deficient conditions. Besides, the expression of *OsMRS2-6* in the young mature leaf was shown to have decreased in the middle stage of Mg starvation (unpublished data). Given that OsMRS2-6 is the chloroplast localizing Mg^2+^ transporter and its gene expression is regulated in linkage with the development of chlorophyll [77], the Mg deficiency might down-regulate the *OsMRS2-6* expression similarly to other chlorophyll-related genes (see Section 2).

## 6. Conclusions and Perspectives

The characteristic function and operation of the CorA transporter has been uncovered steadily in the last decade. Nevertheless, our knowledge about plant Mg^2+^ transporters is still insufficient. The role of plant MRS2/MGT transporters has not been fully clarified yet, and any transporters participating in long-distance Mg transport have not been identified. Additionally, considering the plant as a multicellular organism consists of several kinds of tissue, it could be reasonable to assume the participation of several transporters other than those mentioned in this review in the control of Mg homeostasis in plants. This possibility is also deduced from the fact that at least 35 molecules have been supposed to mediate the potassium fluxes across the membrane [94], and the candidates for the potassium transporters are even increasing [95]. How the Mg deficiency progresses in the leaf has been gradually characterized. During the early-to-mid stage of the Mg deficiency, the leaf antioxidant system is up-regulated as a whole. In the root, the Mg^2+^ uptake rate could be increased under the Mg starved condition. Then, one of the important issues for the future examination could be the determination of the presence or absence of a signaling mechanism linking the different organs in the plant under Mg deficiency.

## References

[1] Marschner H. (1995). Mineral Nutrition of Higher Plants.

[2] Gerendás J., Führs H. (2013). The significance of magnesium for crop quality. Plant Soil.

[3] Hermans C., Conn S.J., Chen J., Xiao Q., Verbruggen N. (2013). An update on magnesium homeostasis mechanisms in plants. Metallomics.

[4] Fischer E.S., Bremer E. (1993). Influence of magnesium deficiency on rates of leaf expansion, starch and sucrose accumulation, and net assimilation in *Phaseolus vulgaris*. Physiol. Plant.

[5] Fischer E.S., Lohaus G., Heineke D., Heldt H.W. (1998). Magnesium deficiency results in accumulation of carbohydrates and amino acids in source and sink leaves of spinach. Physiol. Plant.

[6] Hermans C., Johnson G.N., Strasser R.J., Verbruggen N. (2004). Physiological characterisation of magnesium deficiency in sugar beet: Acclimation to low magnesium differentially affects photosystems I and II. Planta.

[7] Hermans C., Verbruggen N. (2005). Physiological characterization of Mg deficiency in *Arabidopsis thaliana*. J. Exp. Bot..

[8] Kobayashi N.I., Saito T., Iwata N., Ohmae Y., Iwata R., Tanoi K., Nakanishi T.M. (2013). Leaf senescence in rice due to magnesium deficiency mediated defect in transpiration rate before sugar accumulation and chlorosis. Physiol. Plant.

[9] Ding Y., Luo W., Xu G. (2006). Characterisation of magnesium nutrition and interaction of magnesium and potassium in rice. Ann. Appl. Biol..

[10] Hermans C., Vuylsteke M., Coppens F., Cristescu S.M., Harren F.J.M., Inzé D., Verbruggen N. (2010). Systems analysis of the responses to long-term magnesium deficiency and restoration in *Arabidopsis thaliana*. New Phytol..

[11] Fischer E.S. (1997). Photosynthetic irradiance response curves of Phaseolus vulgaris under moderate or severe magnesium deficiency. Photosynthetica.

[12] Sun O.J., Payn T.W. (1999). Magnesium nutrition and photosynthesis in Pinus radiata: Clonal variation and influence of potassium. Tree Physiol..

[13] Cakmak I., Hengeler C., Marschner H. (1994). Partitioning of shoot and root dry matter and carbohydrates in bean plants suffering from phosphorus, potassium and magnesium deficiency. J. Exp. Bot..

[14] Cakmak I., Hengeler C., Marschner H. (1994). Changes in phloem export of sucrose in leaves in response to phosphorus, potassium and magnesium deficiency in bean plants. J. Exp. Bot..

[15] Hermans C.C., Bourgis F.F., Faucher M.M., Strasser R.J.R., Delrot S.S., Verbruggen N.N. (2005). Magnesium deficiency in sugar beets alters sugar partitioning and phloem loading in young mature leaves. Planta.

[16] Marschner H., Cakmak I. (1989). High light intensity enhances chlorosis and necrosis in leaves of zinc, potassium, and magnesium deficient bean (*Phaseolus vulgaris*) plants. J. Plant Physiol..

[17] Cakmak I., Yazici A.M. (2010). Magnesium: A forgotten element in crop production. Better Crops.

[18] Hermans C., Vuylsteke M., Coppens F., Craciun A., Inzé D., Verbruggen N. (2010). Early transcriptomic changes induced by magnesium deficiency in *Arabidopsis thaliana* reveal the alteration of circadian clock gene expression in roots and the triggering of abscisic acid-responsive genes. New Phytol..

[19] Jezek M., Geilfus C.-M., Bayer A., Mühling K.-H. (2014). Photosynthetic capacity, nutrient status, and growth of maize (Zea mays L.) upon MgSO_4_ leaf-application. Front. Plant Sci..

[20] Pei Z.-M., Murata Y., Benning G., Thomine S., Klüsener B., Allen G.J., Grill E., Schroeder J.I. (2000). Calcium channels activated by hydrogen peroxide mediate abscisic acid signalling in guard cells. Nature.

[21] Tewari R.K., Kumar P., Sharma P.N. (2006). Magnesium deficiency induced oxidative stress and antioxidant responses in mulberry plants. Sci. Hortic..

[22] Cakmak I., Marschner H. (1992). Magnesium deficiency and high light intensity enhance activities of superoxide dismutase, ascorbate peroxidase, and glutathione reductase in bean leaves. Plant Physiol..

[23] Chou T.-S., Chao Y.-Y., Huang W.-D., Hong C.-Y., Kao C.H. (2011). Effect of magnesium deficiency on antioxidant status and cadmium toxicity in rice seedlings. J. Plant Physiol..

[24] Hermans C., Chen J., Coppens F., Inzé D., Verbruggen N. (2011). Low magnesium status in plants enhances tolerance to cadmium exposure. New Phytol..

[25] Yang G.-H., Yang L.-T., Jiang H.-X., Li Y., Wang P., Chen L.-S. (2012). Physiological impacts of magnesium-deficiency in Citrus seedlings: Photosynthesis, antioxidant system and carbohydrates. Trees.

[26] Wang H., Ma F., Cheng L. (2010). Metabolism of organic acids, nitrogen and amino acids in chlorotic leaves of “Honeycrisp” apple (*Malus domestica Borkh*) with excessive accumulation of carbohydrates. Planta.

[27] Yang L.T., Yang G.H., You X., Zhou C.P., Lu Y.B., Chen L.S. (2013). Magnesium deficiency-induced changes in organic acid metabolism of *Citrus sinensis* roots and leaves. Biol. Plant.

[28] Li Z., Philip D., Neuhaeuser B., Schulze W.X., Ludewig U. (2015). Protein dynamics in young maize root hairs in response to macro- and micro-nutrient deprivation. J. Proteome Res..

[29] Conn S.J., Conn V., Tyerman S.D., Kaiser B.N., Leigh R.A., Gilliham M. (2011). Magnesium transporters, MGT2/MRS2-1 and MGT3/MRS2-5, are important for magnesium partitioning within *Arabidopsis thaliana* mesophyll vacuoles. New Phytol..

[30] Kamiya T., Yamagami M., Hirai M.Y., Fujiwara T. (2012). Establishment of an *in planta* magnesium monitoring system using CAX3 promoter-luciferase in *Arabidopsis*. J. Exp. Bot..

[31] Lenz H., Dombinov V., Dreistein J., Reinhard M.R., Gebert M., Knoop V. (2013). Magnesium deficiency phenotypes upon multiple knockout of *Arabidopsis thaliana* MRS2 Clade B genes can be ameliorated by concomitantly reduced calcium supply. Plant Cell Physiol..

[32] Hmiel S.P., Snavely M.D., Florer J.B., Maguire M.E., Miller C.G. (1989). Magnesium transport in *Salmonella typhimurium*: Genetic characterization and cloning of three magnesium transport loci. J. Bacteriol..

[33] Eshaghi S., Niegowski D., Kohl A., Martinez Molina D., Lesley S.A., Nordlund P. (2006). Crystal structure of a divalent metal ion transporter CorA at 2.9 angstrom resolution. Science.

[34] Maguire M.E. (1992). MgtA and MgtB: Prokaryotic P-type ATPases that mediate Mg^2+^ influx. J. Bioenerg. Biomembr..

[35] Tao T., Snavely M.D., Farr S.G., Maguire M.E. (1995). Magnesium transport in *Salmonella typhimurium*: *mgtA* encodes a P-type ATPase and is regulated by Mg^2+^ in a manner similar to that of the *mgtB* P-type ATPase. J. Bacteriol..

[36] Snavely M.D., Miller C.G., Maguire M.E. (1991). The *mgtB* Mg^2+^ transport locus of *Salmonella typhimurium* encodes a P-type ATPase. J. Biol. Chem..

[37] Snavely M.D., Florer J.B., Miller C.G., Maguire M.E. (1989). Magnesium transport in *Salmonella typhimurium*: Expression of cloned genes for three distinct Mg^2+^ transport systems. J. Bacteriol..

[38] Smith R.L., Thompson L.J., Maguire M.E. (1995). Cloning and characterization of MgtE, a putative new class of Mg^2+^ transporter from *Bacillus firmus* OF4. J. Bacteriol..

[39] Chamnongpol S., Groisman E.A. (2002). Mg^2+^ homeostasis and avoidance of metal toxicity. Mol. Microbiol..

[40] Dann C.E., Wakeman C.A., Sieling C.L., Baker S.C., Irnov I., Winkler W.C. (2007). Structure and mechanism of a metal-sensing regulatory RNA. Cell.

[41] Wabakken T., Rian E., Kveine M., Aasheim H.-C. (2003). The human solute carrier SLC41A1 belongs to a novel eukaryotic subfamily with homology to prokaryotic MgtE Mg^2+^ transporters. Biochem. Biophys. Res. Commun..

[42] Snavely M.D., Florer J.B., Miller C.G., Maguire M.E. (1989). Magnesium transport in Salmonella typhimurium: ^28^Mg^2+^ transport by the CorA, MgtA, and MgtB systems. J. Bacteriol..

[43] MacDiarmid C.W., Gardner R.C. (1998). Overexpression of the *Saccharomyces cerevisiae* magnesium transport system confers resistance to aluminum ion. J. Biol. Chem..

[44] Bui D.M., Gregan J., Jarosch E., Ragnini A., Schweyen R.J. (1999). The bacterial magnesium transporter CorA can functionally substitute for its putative homologue Mrs2p in the yeast inner mitochondrial membrane. J. Biol. Chem..

[45] Pisat N.P., Pandey A., MacDiarmid C.W. (2009). MNR2 regulates intracellular magnesium storage in *Saccharomyces cerevisiae*. Genetics.

[46] Knoop V., Groth-Malonek M., Gebert M., Eifler K., Weyand K. (2005). Transport of magnesium and other divalent cations: Evolution of the 2-TM-GxN proteins in the MIT superfamily. Mol. Genet. Genom..

[47] Szegedy M.A.M., Maguire M.E.M. (1999). The CorA Mg^2+^ transport protein of *Salmonella typhimurium*. Mutagenesis of conserved residues in the second membrane domain. J. Biol. Chem..

[48] Sponder G., Svidovà S., Khan M.B., Kolisek M., Schweyen R.J., Carugo O., Djinović-Carugo K. (2013). The G-M-N motif determines ion selectivity in the yeast magnesium channel Mrs2p. Metallomics.

[49] Dalmas O., Sandtner W., Medovoy D., Frezza L., Bezanilla F., Perozo E. (2014). A repulsion mechanism explains magnesium permeation and selectivity in CorA. Proc. Natl. Acad. Sci. USA.

[50] Dalmas O., Sompornpisut P., Bezanilla F., Perozo E. (2014). Molecular mechanism of Mg^2+^-dependent gating in CorA. Nat. Commun..

[51] Guskov A., Nordin N., Reynaud A., Engman H., Lundbäck A.-K., Jong A.J.O., Cornvik T., Phua T., Eshaghi S. (2012). Structural insights into the mechanisms of Mg^2+^ uptake, transport, and gating by CorA. Proc. Natl. Acad. Sci. USA.

[52] Pfoh R., Li A., Chakrabarti N., Payandeh J., Pomès R., Pai E.F. (2012). Structural asymmetry in the magnesium channel CorA points to sequential allosteric regulation. Proc. Natl. Acad. Sci. USA.

[53] Neale C., Chakrabarti N., Pomorski P., Pai E.F., Pomès R. (2015). Hydrophobic gating of ion permeation in magnesium channel CorA. PLoS Comput. Biol..

[54] Hattori M., Tanaka Y., Fukai S., Ishitani R., Nureki O. (2007). Crystal structure of the MgtE Mg^2+^ transporter. Nature.

[55] Hattori M., Iwase N., Furuya N., Tanaka Y., Tsukazaki T., Ishitani R., Maguire M.E., Ito K., Maturana A., Nureki O. (2009). Mg^2+^-dependent gating of bacterial MgtE channel underlies Mg^2+^ homeostasis. EMBO J..

[56] Graschopf A., Stadler J.A., Hoellerer M.K., Eder S., Sieghardt M., Kohlwein S.D., Schweyen R.J. (2001). The yeast plasma membrane protein Alr1 controls Mg^2+^ homeostasis and is subject to Mg^2+^-dependent control of its synthesis and degradation. J. Biol. Chem..

[57] Lim P.H., Pisat N.P., Gadhia N., Pandey A., Donovan F.X., Stein L., Salt D.E., Eide D.J., MacDiarmid C.W. (2011). Regulation of Alr1 Mg transporter activity by intracellular magnesium. PLoS ONE.

[58] Horie T., Brodsky D.E., Costa A., Kaneko T., Schiavo F.L., Katsuhara M., Schroeder J.I. (2011). K^+^ transport by the OsHKT2;4 transporter from rice with atypical Na^+^ transport properties and competition in permeation of K^+^ over Mg^2+^ and Ca^2+^ ions. Plant Physiol..

[59] Pottosin I.I., Tikhonova L.I., Hedrich R., Schönknecht G. (1997). Slowly activating vacuolar channels can not mediate Ca^2+^-induced Ca^2+^ release. Plant J..

[60] Guo K.M., Babourina O., Christopher D.A., Borsic T., Rengel Z. (2010). The cyclic nucleotide-gated channel AtCNGC10 transports Ca^2+^ and Mg^2+^ in *Arabidopsis*. Physiol. Plant.

[61] Demidchik V., Maathuis F.J.M. (2007). Physiological roles of nonselective cation channels in plants: From salt stress to signalling and development. New Phytol..

[62] Shaul O., Hilgemann D.W., de-Almeida-Engler J., van Montagu M., Inz D., Galili G. (1999). Cloning and characterization of a novel Mg^2+^/H^+^ exchanger. EMBO J..

[63] David-Assael O., Saul H., Saul V., Mizrachy-Dagri T., Berezin I., Brook E., Shaul O. (2005). Expression of AtMHX, an *Arabidopsis* vacuolar metal transporter, is repressed by the 5′ untranslated region of its gene. J. Exp. Bot..

[64] Gaash R., Elazar M., Mizrahi K., Avramov-Mor M., Berezin I., Shaul O. (2013). Phylogeny and a structural model of plant MHX transporters. BMC Plant Biol..

[65] Borrelly G., Boyer J.C., Touraine B., Szponarski W., Rambier M., Gibrat R. (2001). The yeast mutant vps5Δ affected in the recycling of Golgi membrane proteins displays an enhanced vacuolar Mg^2+^/H^+^ exchange activity. Proc. Natl. Acad. Sci. USA.

[66] Schock I., Gregan J., Steinhauser S., Schweyen R., Brennicke A., Knoop V. (2000). A member of a novel *Arabidopsis thaliana* gene family of candidate Mg^2+^ ion transporters complements a yeast mitochondrial group II intron-splicing mutant. Plant J..

[67] Li L., Tutone A.F., Drummond R.S., Gardner R.C., Luan S. (2001). A novel family of magnesium transport genes in *Arabidopsis*. Plant Cell.

[68] Chen J., Li L.-G., Liu Z.-H., Yuan Y.-J., Guo L.-L., Mao D.-D., Tian L.-F., Chen L.-B., Luan S., Li D.-P. (2009). Magnesium transporter AtMGT9 is essential for pollen development in *Arabidopsis*. Cell Res..

[69] Li L.-G., Sokolov L.N., Yang Y.-H., Li D.-P., Ting J., Pandy G.K., Luan S. (2008). A mitochondrial magnesium transporter functions in *Arabidopsis* pollen development. Mol. Plant.

[70] Mao D.-D., Tian L.-F., Li L.-G., Chen J., Deng P.-Y., Li D.-P., Luan S. (2008). AtMGT7: An *Arabidopsis* gene encoding a low-affinity magnesium transporter. J. Integr. Plant Biol..

[71] Drummond R.S.M., Tutone A., Li Y.-C., Gardner R.C. (2006). A putative magnesium transporter AtMRS2-11 is localized to the plant chloroplast envelope membrane system. Plant Sci..

[72] Gebert M., Meschenmoser K., Svidovà S., Weghuber J., Schweyen R., Eifler K., Lenz H., Weyand K., Knoop V. (2009). A root-expressed magnesium transporter of the MRS2/MGT gene family in *Arabidopsis* thaliana allows for growth in low-Mg^2+^ environments. Plant Cell.

[73] Ishijima S., Shigemi Z., Adachi H., Makinouchi N., Sagami I. (2012). Functional reconstitution and characterization of the *Arabidopsis* Mg^2+^ transporter AtMRS2-10 in proteoliposomes. Biochim. Biophys. Acta.

[74] Deng W., Luo K., Li D., Zheng X., Wei X., Smith W., Thammina C., Lu L., Li Y., Pei Y. (2006). Overexpression of an *Arabidopsis* magnesium transport gene, AtMGT1, in Nicotiana benthamiana confers Al tolerance. J. Exp. Bot..

[75] Visscher A.M., Paul A.-L., Kirst M., Guy C.L., Schuerger A.C., Ferl R.J. (2010). Growth performance and root transcriptome remodeling of *Arabidopsis* in response to mars-like levels of magnesium sulfate. PLoS ONE.

[76] Mao D., Chen J., Tian L., Liu Z., Yang L., Tang R., Li J., Lu C., Yang Y., Shi J. (2014). *Arabidopsis* transporter MGT6 mediates magnesium uptake and is required for growth under magnesium limitation. Plant Cell.

[77] Saito T., Kobayashi N.I., Tanoi K., Iwata N., Suzuki H., Iwata R., Nakanishi T.M. (2013). Expression and functional analysis of the CorA-MRS2-ALR-type magnesium transporter family in rice. Plant Cell Physiol..

[78] Kolisek M., Zsurka G., Samaj J., Weghuber J., Schweyen R.J., Schweigel M. (2003). Mrs2p is an essential component of the major electrophoretic Mg^2+^ influx system in mitochondria. EMBO J..

[79] Wan Q., Ahmad M.F., Fairman J., Gorzelle B., La Fuente M.D., Dealwis C., Maguire M.E. (2011). X-ray crystallography and isothermal titration calorimetry studies of the *Salmonella* zinc transporter ZntB. Structure.

[80] Chen Z.C., Yamaji N., Motoyama R., Nagamura Y., Ma J.F. (2012). Up-regulation of a magnesium transporter gene OsMGT1 is required for conferring aluminum tolerance in rice. Plant Physiol..

[81] Yamaji N., Huang C.F., Nagao S., Yano M., Sato Y., Nagamura Y., Ma J.F. (2009). A Zinc finger transcription factor ART1 regulates multiple genes implicated in aluminum tolerance in rice. Plant Cell.

[82] Chen Z.C., Ma J.F. (2013). Magnesium transporters and their role in Al tolerance in plants. Plant Soil..

[83] Werner T., Nehnevajova E., Köllmer I., Novák O., Strnad M., Krämer U., Schmülling T. (2010). Root-specific reduction of cytokinin causes enhanced root growth, drought tolerance, and leaf mineral enrichment in *Arabidopsis* and tobacco. Plant Cell.

[84] Zhong W., Schobert C., Komor E. (1993). Transport of magnesium ions in the phloem of *Ricinus communis* L. seedlings. Planta.

[85] Hayashi H., Chino M. (1986). Collection of pure phloem sap from wheat and its chemical composition. Plant Cell Physiol..

[86] Kobayashi N.I., Iwata N., Saito T., Suzuki H., Iwata R., Tanoi K., Nakanishi T.M. (2013). Application of ^28^Mg for characterization of Mg uptake in rice seedling under different pH conditions. J. Radioanal. Nucl. Chem..

[87] Kobayashi N.I., Iwata N., Saito T., Suzuki H., Iwata R., Tanoi K., Nakanishi T.M. (2013). Different magnesium uptake and transport activity along the rice root axis revealed by ^28^Mg tracer experiments. Soil Sci. Plant Nutr..

[88] Tanoi K., Kobayashi N., Saito T., Iwata N., Kamada R., Iwata R., Suzuki H., Hirose A., Ohmae Y., Sugita R. (2014). Effects of magnesium deficiency on magnesium uptake activity of rice root, evaluated using ^28^Mg as a tracer. Plant Soil.

[89] Sugita R., Kobayashi N.I., Saito T., Hirose A., Iwata R., Tanoi K., Nakanishi T.M. (2014). Quantitative analysis of ^28^Mg in *Arabidopsis* using real-time radioisotope imaging system (RRIS). Radioisotopes.

[90] Kanno S., Yamawaki M., Ishibashi H., Kobayashi N.I., Hirose A., Tanoi K., Nussaume L., Nakanishi T.M. (2012). Development of real-time radioisotope imaging systems for plant nutrient uptake studies. Philos. Trans. R. Soc. B.

[91] Bose J., Babourina O., Shabala S., Rengel Z. (2013). Low-pH and aluminum resistance in *Arabidopsis* correlates with high cytosolic magnesium content and increased magnesium uptake by plant roots. Plant Cell Physiol..

[92] Schmitz J., Tierbach A., Lenz H., Meschenmoser K., Knoop V. (2013). Membrane protein interactions between different *Arabidopsis thaliana* MRS2-type magnesium transporters are highly permissive. Biochim. Biophys. Acta.

[93] Payandeh J., Li C., Ramjeesingh M., Poduch E., Bear C.E., Pai E.F. (2008). Probing structure-function relationships and gating mechanisms in the CorA Mg^2+^ transport system. J. Biol. Chem..

[94] Mäser P., Thomine S., Schroeder J.I., Ward J.M., Hirschi K., Sze H., Talke I.N., Amtmann A., Maathuis F.J., Sanders D. (2001). Phylogenetic relationships within cation transporter families of *Arabidopsis*. Plant Physiol..

[95] Szczerba M.W., Britto D.T., Kronzucker H.J. (2009). K^+^ transport in plants: Physiology and molecular biology. J. Plant Physiol..

